# Grapefruit Seed Extract as a Natural Derived Antibacterial Substance against Multidrug-Resistant Bacteria

**DOI:** 10.3390/antibiotics10010085

**Published:** 2021-01-18

**Authors:** Hee-Won Han, Jin-Hwan Kwak, Tae-Su Jang, Jonathan Campbell Knowles, Hae-Won Kim, Hae-Hyoung Lee, Jung-Hwan Lee

**Affiliations:** 1 Institute of Tissue Regeneration Engineering (ITREN), Dankook University, Cheonan, Chungcheongnam-do 31116, Korea; 12201075@dankook.ac.kr (H.-W.H.); j.knowles@ucl.ac.uk (J.C.K.); kimhw@dku.edu (H.-W.K.); 2 Department of Biomaterials Science, College of Dentistry, Dankook University, Cheonan, Chungcheongnam-do 31116, Korea; 3 Department of Life Science, Handong Global University, 558 Handong-ro, Pohang, Gyeongsangbuk-do 37554, Korea; jhkwak@handong.edu; 4 Department of Pre-Medi, College of Medicine, Dankook University, 119 Dandae-ro, Cheonan, Chungcheongnam-do 31116, Korea; jangts@dankook.ac.kr; 5 UCL Eastman-Korea Dental Medicine Innovation Centre, Dankook University, 119 Dandae-ro, Cheonan, Chungcheongnam-do 31116, Korea; 6 Division of Biomaterials and Tissue Engineering, Eastman Dental Institute, University College London, Rowland Hill Street, London NW3 2PF, UK; 7 Department of Nanobiomedical Science and BK21 PLUS NBM Global Research Center for Regenerative Medicine, Dankook University, Cheonan, Chungcheongnam-do 31116, Korea; 8 Cell & Matter Institute, Dankook University, Cheonan, Chungcheongnam-do 31116, Korea; 9 Department of Regenerative Dental Medicine, College of Dentistry, Dankook University, Cheonan, Chungcheongnam-do 31116, Korea

**Keywords:** multidrug-resistant bacteria, nosocomial infection, plant extract, grapefruit seed extract, antibacterial activity

## Abstract

Multidrug-resistant (MDR) bacteria are increasing due to the abuse and misuse of antibiotics, and nosocomial infections by MDR bacteria are also increasing. The aim of this study was to identify new substances that can target MDR bacteria among 12 plant extracts that are known to have antibacterial effects. The experiments were performed by the disk diffusion test and microdilution minimum inhibitory concentration (MIC) test, as described by the Clinical and Laboratory Standards Institute (CLSI). By screening against methicillin-sensitive *Staphylococcus aureus* (MSSA), grapefruit seed extract (GSE) was selected from 12 plant extracts for subsequent experiments. GSE showed antibacterial effects against methicillin-resistant *S. aureus* (MRSA) and vancomycin-resistant *S. aureus* (VRSA) in the disk diffusion test. Even at the lowest concentration, GSE showed antibacterial activity in the microdilution MIC test. As a result, we can conclude that GSE is a naturally derived antibacterial substance that exhibits a favorable antibacterial effect even at a very low concentration, so it is a good candidate for a natural substance that can be used to prevent or reduce nosocomial infections as coating for materials used in medical contexts or by mixing a small amount with other materials.

## 1. Introduction

The number of bacterial strains that are resistant to antibiotics continues to increase due to the misuse and abuse of antibiotics. Among these, bacteria that are resistant to several antibiotics are called multidrug-resistant (MDR) bacteria. For example, methicillin-resistant *Staphylococcus aureus* (MRSA), which was first identified in the early 1960s [1], is resistant to other beta-lactam antibiotics, including those in the penicillin class and cephalosporin class, in addition to methicillin [2]. MDR bacteria constitute the leading cause of nosocomial infection, and outcomes in patients infected with MDR bacteria tend to be worse than for those infected by more sensitive organisms [3,4,5]. In addition, the mortality rate from MDR bacteria such as MRSA is significantly higher than for susceptible strains [6]. Therefore, efforts to discover new substances that can target MDR bacteria have become increasingly important.

Antibacterial effects are being studied in various fields, such as tissue engineering [7,8,9], and many experiments have been conducted to identify new substances that can target antibiotic-resistant bacteria. Recently, antimicrobial activity experiments using nanoparticles (NPs) have been actively conducted. The antibacterial effects of nanoparticles such as silver, gold, and ZnO have already been confirmed for over a decade and are still actively being studied [10,11,12,13,14]. In addition, these nanoparticles have also shown antibacterial effects against MDR bacteria such as vancomycin-resistant *Enterococcus* (VRE) and MRSA [15,16,17,18,19]. In addition to nanoparticles, interest in natural products as substitutes for traditional antibiotics to fight multidrug-resistant pathogens has greatly increased [20]. The antibacterial effect of honeydew honey and the antibacterial effects of plant extracts such as black pepper extract, grapefruit seed extract (GSE), and coral *Hibiscus* extract have been proven in many papers [21,22,23,24,25,26,27].

Natural products have long been used as the basis for treatment [28]. Most of the drugs on the market today are natural-based products or their derivatives [29], suggesting that natural-based products may be better accepted by the body and more successful than synthetic chemicals [30]. Therefore, we focused on natural products, especially plant extracts.

In this study, 12 plant extracts known for having antibacterial activity were analyzed by disk diffusion test against MSSA. From this screening test, only GSE showed antibacterial effect. Therefore, the purpose of this study was to evaluate the antibacterial activity of GSE against MDR bacteria such as MRSA and VRSA, which cause serious problems through nosocomial infection, and investigate the cause of the antibacterial effect of GSE.

## 2. Materials and Methods

### 2.1. Bacterial Strains and Culture Conditions

*S. aureus* ATCC6538 (MSSA) and *S. aureus* ATCC33591 (MRSA) were purchased from American Type Culture Collection (ATCC; Manassas, VA, USA). *S. aureus* CCARM3795 (MRSA) was purchased from the Culture Collection of Antimicrobial Resistant Microbes (Nowon-gu, Seoul, Korea). VRSA48, which was clinically isolated, was generously provided by the Korea Research Institute of Bioscience & Biotechnology (KRIBB, Yuseong-gu, Daejeon, Korea) through Prof. Jin-Hwan Kwak of Handong Global University (HGU, Buk-gu, Pohang, Korea). All strains, which had been kept as glycerol stock solutions in a −80 °C deep freezer, were streaked on tryptic soy agar (TSA, Difco Laboratories, Becton Dickinson, Sparks, MD, USA) plates and incubated at 35 °C ± 2 °C for 18 h. A single colony, after incubation, was transferred to tryptic soy broth (TSB, Difco Laboratories, Sparks, MD, USA) and incubated with shaking at 35 °C ± 2 °C for 18 h. To achieve the desired concentration, dilutions were performed with phosphate-buffered saline (PBS; Gibco, Grand Island, NY, USA) for the disk diffusion test and TSB media for the microdilution minimum inhibitory concentration (MIC) test.

### 2.2. Plant Extracts and Antimicrobial Agents

Among the natural extracts already used in soaps and cosmetics on the market, but not applicable for biomedical settings now, twelve extracts that have antibacterial activity and are considered economical were purchased and used. Bamboo extract was purchased from KoreaSimilac (Pocheon, Gyeonggi-do, Korea). Refined wood vinegar, rosemary, *Pinus densiflora* leaf, *Sophora*, *Cinnamomum cassia* bark, *Hibiscus sabdariffa* flower, *Chamomilla recutita* (*Matricaria*) flower, *Centella asiatica*, *Houttuynia cordata*, and Yucca extracts were purchased from HERBFLORA (Dobong-gu, Seoul, Korea). Grapefruit seed extract (GSE) was purchased from CANDLEIKEA (Jung-gu, Seoul, Korea). Bamboo, rosemary, *Sophora*, *Centella asiatica*, *Houttuynia cordata*, and Yucca extracts were extracted by hot water extraction. *Cinnamomum cassia* bark, *Hibiscus sabdariffa* flower, and *Chamomilla recutita* (*Matricaria*) flower extracts were extracted by low temperature extraction. The extraction types for refined wood vinegar, *Pinus densiflora leaf*, and Grapefruit seed extracts are unrevealed due to the company’s confidentiality. Vancomycin (VAN), oxacillin (OXA), and linezolid (LZD) were purchased from Sigma-Aldrich (St. Louis, MO, USA).

### 2.3. pH Measurements

The pH of GSE, 76% G (76% glycerol), and pH 76% G (pH-adjusted 76% glycerol) were measured with a pH meter (inoLab pH 7110, WTW, Weilheim, Germany). The 76% glycerol solution was prepared by adding distilled water (DW) to glycerol (≥99.0%, Sigma-Aldrich, St. Louis, MO, USA), and pH 76% G was derived from 76% G using acetic acid. The electrode was soaked in each solution at room temperature (24 °C), and each measurement was repeated three times and averaged.

### 2.4. GSE Analysis by LC-MS and LC-MS/MS

GSE was analyzed by liquid chromatography/mass spectrometry (LC-MS) and liquid chromatography/tandem mass spectrometry (LC-MS/MS) with an Ultimate 3000 RS-Q-Exactive Orbitrap Plus (Thermo Fisher Scientific, Waltham, MA, USA) at the Yonsei Center for Research Facilities (YCRF, Seodaemun-gu, Seoul, Korea). For the negative-mode LC condition, an Acquity UPLC BEH C18 (1.7 µm, 2.1 × 100 mm) was used as an LC column at 40 °C. The injection volume was 3 µL. The run time was 12 min. The mobile phase consisting of solvent A, 6.5 mM ammonium bicarbonate in DW, and solvent B, 6.5 mM ammonium bicarbonate in acetonitrile (ACN) was delivered at a flow rate of 0.4 mL/min. The following linear gradient was used: 0 min, 0% B; 1 min, 10% B; 9 min, 100% B; 12 min; 10% B. The electrospray ionization (ESI) (negative ionization mode) conditions were capillary voltage was 3.0 kV, S-lens RF level was 45, capillary temperature was 370 °C, and aux gas heater temperature was 285 °C. The sheath and aux gas flows were 60 and 20, respectively.

### 2.5. In Vitro Studies

#### 2.5.1. Disk Diffusion Test

All strains that were grown on TSA plates at 35 °C ± 2 °C for 18 h were subcultured separately into 3 mL of TSB media at 35 °C ± 2 °C for 18 h. The cultured bacteria were diluted using PBS to obtain bacterial cell densities of approximately 1~2 × 10^8^ CFU (colony forming unit)/mL, and then the diluted bacterial suspensions were spread using sterilized cotton swabs on TSA plates (100 mm × 15 mm). Bacterial concentration was determined by measuring the optical density at 600 nm with a spectrophotometer. The actual number of colonies was confirmed by diluting the bacterial culture solution that was diluted to the desired concentration to 10^3^ CFU/mL, dropping 100 µL on an MHA plate, spreading and incubating. After allowing the surface of each medium to dry for 3–5 min, ADVANTEC paper disks (8 mm/0.7 mm) that had been sterilized with ethylene gas were placed on TSA plates and pressed with forceps to ensure complete contact with each agar surface. For the screening test, 20 µL of plant extract and linezolid (1.5 mg/mL) were loaded onto paper disks on agar plates spread with *S. aureus* ATCC6538 (MSSA). To check the antibacterial effect of GSE on MDR bacteria, 20 µL of GSE and linezolid (1.5 mg/mL) were loaded onto paper disks on separate agar plates spread with MSSA and MDR bacteria. To investigate the effects of glycerol and pH on bacterial growth, 20 µL of GSE, 76% G, pH 76% G and linezolid (1.5 mg/mL) were loaded separately onto paper disks on plates spread with MSSA or MDR bacteria, and then agar plates were incubated at 35 °C ± 2 °C for 18 h. After incubation, the diameters of the inhibition zones were measured from edge to edge across the centers of the disks. All experiments were conducted 3 times.

#### 2.5.2. Microdilution MIC Test

The MICs of GSE, 76% G, pH 76% G, pH 76% G with naringin (1.7 mg/mL), and antimicrobial agents were determined using the twofold microdilution broth method. Naringin was dissolved in DMSO first and then diluted with pH 76% G, and all other test compounds were diluted with DW. GSE, 76% G, pH 76% G, and pH 76% G with naringin (1.7 mg/mL) were diluted to yield 10 concentrations from undiluted solution to 1/512, and antimicrobial agents were diluted to yield 10 concentrations from 64 µg to 0.125 µg/mL. All strains that were grown on TSA plates at 35 °C ± 2 °C for 18 h were subcultured into 3 mL of TSB media at 35 °C ± 2 °C for 18 h. The cultured bacterial broth suspensions were diluted using TSB media to obtain bacterial cell densities of approximately 1 × 10^6^ CFU/mL. One-hundred microliters of each diluted bacterial suspension were seeded in 96-well plates containing 100 µL of serially diluted test compounds to achieve a bacterial concentration of 5 × 10^5^ CFU/well, which also resulted in 1/2 dilution of test compounds. The 96-well plates were incubated at 35 °C ± 2 °C for 18 h. MICs were defined as the lowest concentrations that completely inhibited the growth of bacteria when viewed with the unaided eye. As the MIC test was performed by finding the MIC values of the antibiotics for each bacterium several times, each MIC test including GSE was completed in one experiment.

### 2.6. Statistical Analysis

All data are shown as mean ± SD. In each experiment, n is the number of repeated trials. Statistical significance was determined by unpaired, two-tailed t-tests for differences between two groups and using one-way ANOVA and Dunnett’s multiple comparisons tests for differences among more than two groups. GraphPad Prism 8 software (San Diego, CA, USA) was used.

## 3. Results and Discussion

### 3.1. Antibacterial Effect of GSE in Disk Diffusion Test

**Figure 1:**
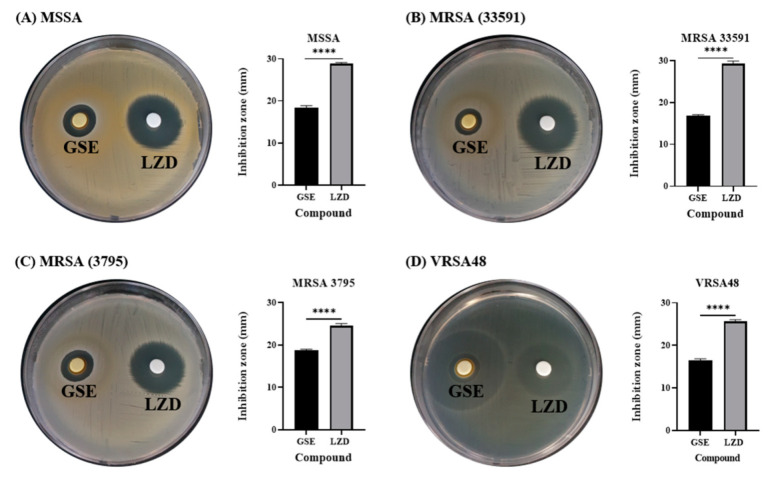
Disk diffusion tests and inhibition zone diameters for Grapefruit seed extract (GSE) and linezolid (LZD) against methicillin-sensitive *Staphylococcus aureus* (MSSA), methicillin-resistant *S. aureus* (MRSA), and VRSA. (**A**) *S. aureus* ATCC 6538 (MSSA), (**B**) *S. aureus* ATCC 33591 (MRSA), (**C**) *S. aureus* CCARM 3795 (MRSA), and (**D**) vancomycin-resistant *S. aureus* (VRSA48). The concentration of LZD was 30 µg/disk, following the CLSI guidelines. The diameters of the inhibition zones for GSE and LZD are illustrated by the bar graphs as the means ± SD (*n* = 3). All tested bacteria were susceptible to LZD, according to the CLSI guidelines (≥21 mm for *Staphylococcus* spp.), and GSE also showed antibacterial effects against all tested bacteria. *p* values were calculated using two-tailed, unpaired *t*-tests in this figure. **** *p* < 0.0001.

Among the commercially available plant extracts, 12 types of extracts that are known to have antibacterial effects were purchased, and the antibacterial activities of 12 extracts (specified as numbers in Table S1) were analyzed against MSSA ATCC6538 (Figure S1) by the disk diffusion test. The disk diffusion test is a standard method described by the Clinical and Laboratory Standards Institute (CLSI) and is an experimental method that can quickly and easily evaluate the antibacterial activity of many compounds [31]. If an extract exerts an antibacterial effect, an inhibition zone will form around the paper disk where the extract was dropped. A highly effective antibacterial agent produces a wide ring without bacterial growth, whereas an ineffective antibacterial agent shows no change in the concentration of bacteria around it. Except for GSE, the other plant extracts showed no antibacterial effects against MSSA in the disk diffusion test. For subsequent experiments, therefore, only GSE was used. GSE showed inhibition zones against MDR bacteria (MRSA and VRSA) as well as MSSA (Figure 1). The inhibition zones of GSE against MSSA, MRSA 33591, MRSA 3795, and VRSA48 were 18.3 ± 0.6 mm, 16.8 ± 0.6 mm, 18.8 ± 0.3 mm, and 16.4 ± 0.4 mm, respectively (*n* = 3). In the experiment with VRSA48, there were two circles surrounding the GSE paper disk. One was a small circle (real inhibition zone), and the other was a larger circle (zone of incomplete inhibition of bacterial growth). Therefore, the smaller circle was measured as the inhibition zone. GSE showed antibacterial effects against all tested bacteria. The inhibition zones of LZD, which was used as a control, against MSSA, MRSA 33591, MRSA 3795, and VRSA48, were 28.8 ± 0.3 mm, 29.3 ± 0.6 mm, 24.6 ± 0.5 mm, and 25.7 ± 0.4 mm, respectively (*n* = 3). These diameter values were illustrated with bar graphs. All tested bacteria were susceptible to LZD, according to the CLSI guidelines (≥21 mm for *Staphylococcus* spp.) [31]. Although the inhibition zone for GSE was smaller than that of LZD, the antibacterial effect of GSE was confirmed through a disk diffusion test. In the next experiment, the pH and percentage of the solvent of the GSE were measured because the antibacterial effect of GSE may occur due to the solvent in which the GSE was dissolved or the acidic properties of the GSE itself.

### 3.2. GSE Characterization

**Figure 2:**
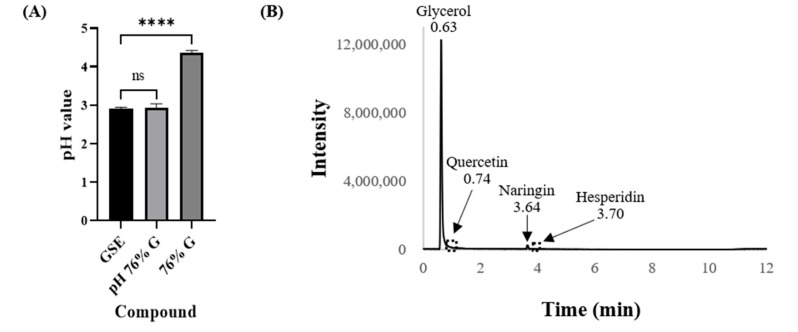
GSE characterization. (**A**) The pH values for GSE, 76% G, and pH 76% G. The pH values of the tested compounds are illustrated by the bar graphs as the means ± SD (*n* = 3). The pH 76% G was prepared from 76% G using acetic acid, and it did not show any significant difference from the pH value of GSE. (**B**) LC-MS/MS results of GSE. Glycerol and naringin were analyzed by LC-MS/MS and LC-MS for quercetin and hesperidin. Because the peaks for quercetin and hesperidin were too small (not visible on the graph), their peaks were replaced by dashed boxes. The numbers above the arrows and peaks represent the retention times. *p* values were calculated using one-way ANOVA with Dunnett’s multiple comparisons test in this figure. ns, not significant; **** *p* < 0.0001.

As the pH or solvent of GSE solutions could affect bacterial growth, the pH of the GSE and the percentage solvent were measured with a pH meter (inoLab pH 7110, Germany) and an LC-MS/MS system (Thermo Fisher Scientific, Waltham, MA, USA), respectively. Glycerol was analyzed quantitatively because we had received information from HERBFLORA (Seoul, Korea) that the GSE extract was dissolved in glycerol that is highly effective for obtaining phenolic compounds [32]. It was confirmed that the pH of GSE was 2.90 ± 0.04 (*n* = 3) and that the GSE extract was dissolved in 76% glycerol (Figure 2). Based on these results, 76% glycerol (76% G throughout the paper) was prepared, and pH-adjusted 76% glycerol (pH 76% G throughout the paper) was prepared from 76% G using acetic acid. The pH of 76% G and pH 76% G were measured using a pH meter (inoLab pH 7110, Germany) and showed pH values of 4.37 ± 0.06 and 2.94 ± 0.01, respectively (*n* = 3). The pH 76% G was prepared carefully without any significant difference from the pH of GSE. The antibacterial effect of GSE is already known, and since it is known that GSE contains flavonoids with antimicrobial effects, flavonoids were also analyzed by LC-MS and LC-MS/MS in addition to glycerol [25,26,33,34,35]. GSE is known to contain relatively large amounts of the flavonoid naringin, so naringin was quantitatively analyzed. Glycerol and naringin were analyzed by LC-MS/MS, and the other flavonoids hesperidin, eriocitrin, poncirin, and quercetin were analyzed by LC-MS. To measure the concentration of glycerol and naringin in GSE, reference compounds glycerol (≥99.0%, Sigma-Aldrich, St. Louis, MO, USA) and naringin (≥95.0%, Sigma-Aldrich, St. Louis, MO, USA) were purchased. These reagents dissolved in DW were measured by LC-MS/MS, and then compared with GSE analysis results. Glycerol concentration was measured at 762.544 mg/mL (it was considered to be 760 mg/mL, 76%) at 0.63 min, and naringin concentration was determined as 1.726 mg/mL (it was considered to be 1.7 mg/mL) at 3.64 min. From the LC-MS results, quercetin and hesperidin were identified at 0.74 min and 3.70 min, respectively, in GSE (Figure S2). However, because the quercetin and hesperidin concentrations were very low, no peaks were seen in Figure 2B. Eriocitrin and poncirin were not detected in GSE.

### 3.3. Antibacterial Effects of Solvent and pH of GSE in Disk Diffusion Tests

**Figure 3:**
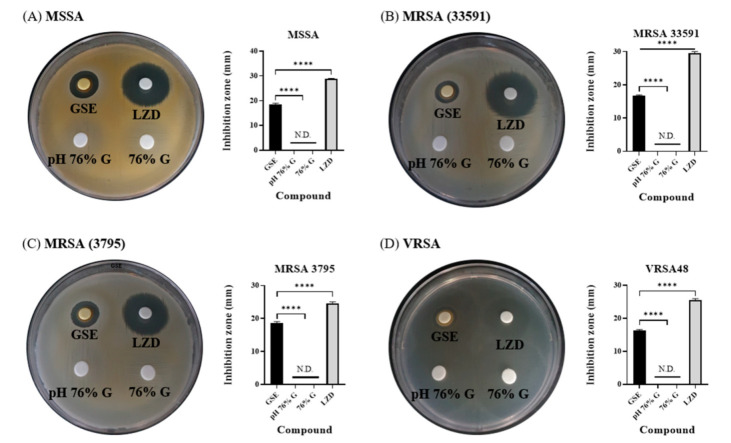
Disk diffusion tests and inhibition zone diameters for the tested compounds and LZD against MSSA, MRSA, and VRSA. (**A**) *S. aureus* ATCC 6538 (MSSA), (**B**) *S. aureus* ATCC 33591 (MRSA), (**C**) *S. aureus* CCARM 3795 (MRSA), and (**D**) VRSA48. The concentration of LZD was 30 µg/disk. N.D. means that an inhibition zone was not detected. The diameters of the inhibition zones for GSE and LZD are illustrated by the bar graphs as means ± SD (*n* = 3). GSE and LZD showed antibacterial effects, as shown in Figure 1, but 76% G and pH 76% G did not show any inhibition zones. This indicates that glycerol and the pH of GSE have little influence on the antibacterial effect of GSE. *p* values were calculated using one-way ANOVA with Dunnett’s multiple comparisons test in this figure. **** *p* < 0.0001.

GSE dissolved in 76% G was analyzed, and it also had a low pH value. Because high concentrations of glycerol and low pH can affect bacterial growth [36,37], the antimicrobial activity of 76% G and pH 76% G was evaluated along with GSE and LZD. In disk diffusion tests, GSE and LZD showed effects almost identical to those shown in Figure 1, but 76% G and pH 76% G did not show any inhibition zones against any tested bacteria (Figure 3). Therefore, 76% G and pH 76% G were marked as N.D. (not detected) in bar graphs. In the experiment with VRSA48, it appeared that an inhibition zone formed in the glycerol group, but bacterial growth was checked inside the circle which resembled an inhibition zone. From disk diffusion tests, we found that glycerol and the pH of GSE have little effect on the antibacterial effect of GSE.

### 3.4. Antibacterial Effects of Solvent and pH of GSE in Microdilution MIC Test

To further investigate whether glycerol or the pH of GSE influenced the antimicrobial activity of GSE, microdilution MIC tests were performed. The MIC test is also a standard method described by the CLSI. This method is more detailed than the disk diffusion test because it shows antibacterial effects according to various concentrations. In the 96-well plate figures, the MICs of pH 76% G and 76% G are indicated by red arrows and the MICs of antibiotics by blue arrows. The MIC of GSE and the MIC for >32 µg/mL antibiotic are not indicated. In MIC tests, GSE showed potent antibacterial activity against all tested bacteria. Even at a concentration of 1/1024, the growth of all tested bacteria was suppressed. The red dots that resemble bacterial colonies at the 1/64 and 1/128 concentrations and the turbidity seen in the other concentration groups were the result of the influence of the GSE itself (Figure S3), and it was confirmed that bacteria did not grow in the 1/2 through the 1/1024 concentrations by checking values of CFU (data not shown). The 76% G and pH 76% G treatments showed slight antibacterial effects in MIC tests. pH 76% G showed a MIC value one level lower (1/4 concentration) than that for 76% G (1/2 concentration), except for MRSA. For MRSA, both 76% G and pH 76% G showed a MIC value at 1/2 concentration. Antibiotics were used as controls to check if the experiment was performed properly, and they were also used to check MRSA and VRSA. MSSA and MRSA can be distinguished through the OXA MIC value (≥4 μg/mL, MRSA), and VRSA can be identified through the VAN MIC value (≥16 μg/mL, VRSA) [31]. VAN, OXA, and LZD showed MIC values of 1 μg/mL, 0.25 μg/mL, and 2 μg/mL against MSSA, respectively. VAN, OXA, and LZD showed MIC values of 1 μg/mL, >32 μg/mL, and 1 μg/mL against MRSA 33591, respectively. VAN, OXA, and LZD showed MIC values of 1 μg/mL, >32 μg/mL, and 1 μg/mL against MRSA 3795, respectively. VAN, OXA, and LZD showed MIC values of >32 μg/mL, >32 μg/mL, and 4 μg/mL against VRSA48, respectively. These results are illustrated as bar graphs. The MICs of GSE, pH 76% G, and 76% G can be read as values of the left *y*-axis (red arrow), and MICs of antibiotics can be read as values of the right *y*-axis (blue arrow). GSE was expressed as <1/1024, as bacteria did not grow even at 1/1024 concentration in any experimental groups. For antibiotics, MIC was expressed as >32 if the growth of bacteria was not inhibited even at a concentration of 32 μg/mL. Based on these antibiotic MIC values, MSSA, MRSA, and VRSA could be identified, and the reliability of the experiment was confirmed. As the MIC test was performed by finding the MIC values of the antibiotics for each bacterial strain several times, each MIC test including that for GSE was completed in one experiment. In disk diffusion tests and MIC tests, it was confirmed that the pH and glycerol did not significantly affect the antimicrobial activity of GSE. This result signifies that there are substances that affect the antibacterial activity of GSE in addition to the pH and solvent of GSE. Several articles related to the antibacterial activity of GSE or other plants have indicated that flavonoids have antibacterial activity [38,39,40]. GSE contained a large amount of the flavonoid naringin, so the following experiment was conducted by focusing on naringin.

### 3.5. Antibacterial Effect of Naringin

**Figure 4:**
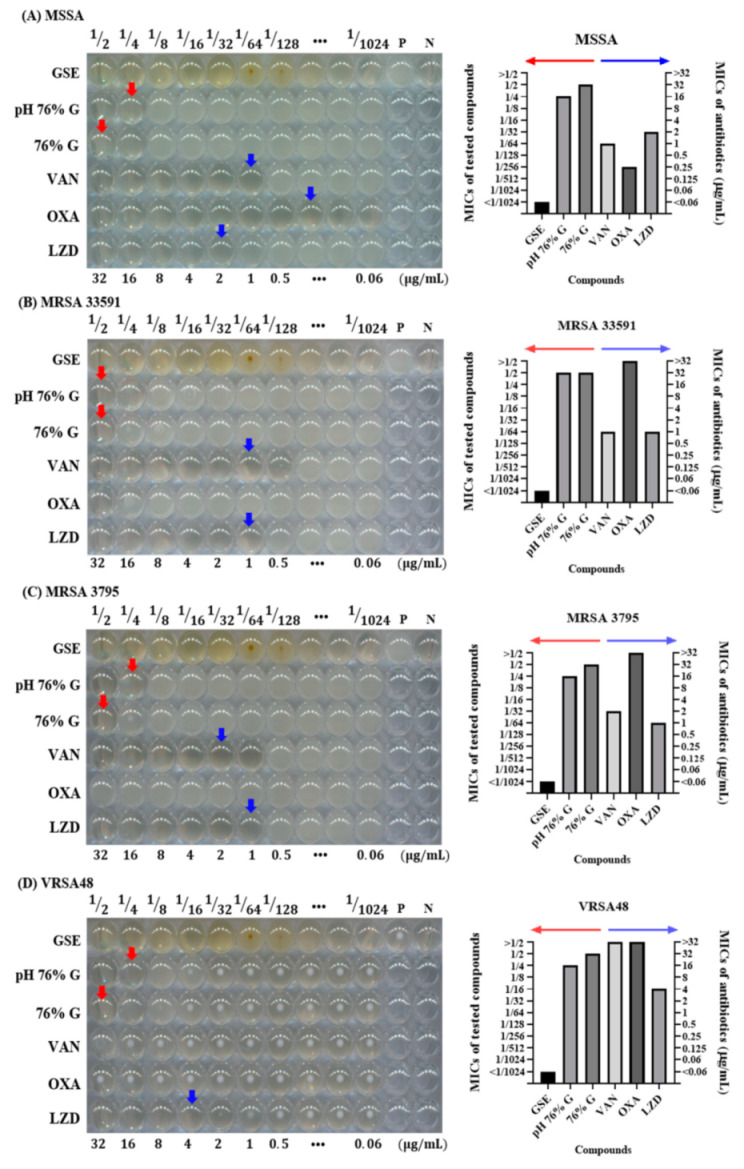
Microdilution minimum inhibitory concentration (MIC) tests for compounds and antibiotics tested against MSSA, MRSA, and VRSA. (**A**) *S. aureus* ATCC 6538 (MSSA), (**B**) *S. aureus* ATCC 33591 (MRSA), (**C**) *S. aureus* CCARM 3795 (MRSA), and (**D**) VRSA48. P is a positive control without any antimicrobial agents, and N is a negative control with only media and DW. In the 96-well plate, from left to right, the tested compound concentrations were 1/2 to 1/1024, and the concentrations of antibiotics were 32 to 0.06 µg/mL. The MICs of pH 76% G and 76% G are indicated by red arrows, and those for antibiotics are indicated by blue arrows. MIC values <1/1024 or >32 µg/mL are not indicated by arrows. The MIC values of the tested compounds and antibiotics for each of the bacterial strains are also illustrated by bar graphs next to the 96-well plate figures. In the bar graph, the MIC values of the tested compounds can be read along the left *y*-axis indicated by the red arrow, and the MIC values of antibiotics along the right *y*-axis are indicated by the blue arrow. MRSA 335591 and 3795 showed OXA MIC values >32 µg/mL, confirmed to be MRSA, and VRSA48 also showed MIC values >32 µg/mL for OXA and VAN, confirming VRSA. The GSE showed MIC values of <1/1024 concentration for all tested bacteria. Excluding MRSA 33591, pH 76% G showed an MIC value (1/4 concentration) lower than 76% G (1/2 concentration), but it was still insufficient to explain the antibacterial effect of GSE.

**Figure 5:**
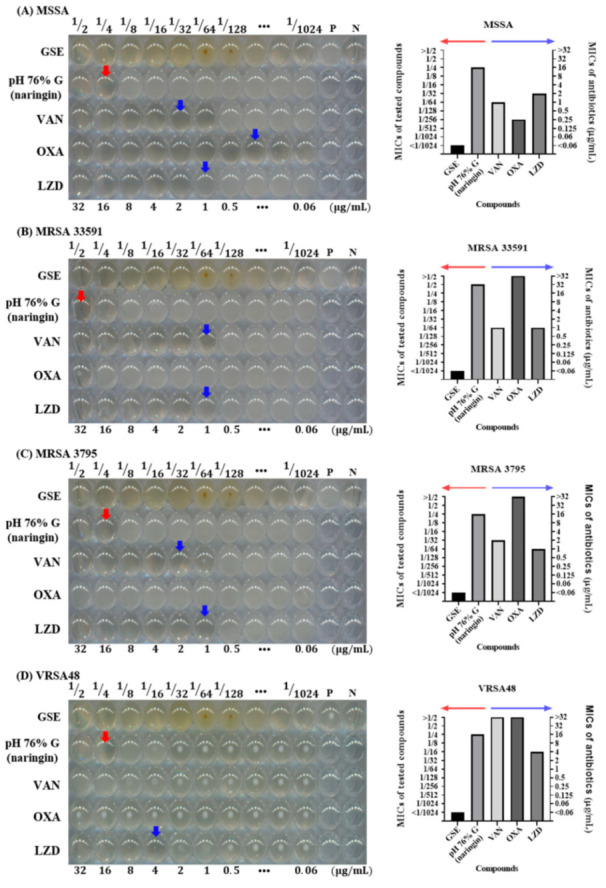
Microdilution MIC test with naringin against MSSA, MRSA, and VRSA. (**A**) *S. aureus* ATCC 6538 (MSSA), (**B**) *S. aureus* ATCC 33591 (MRSA), (**C**) *S. aureus* CCARM 3795 (MRSA), and (**D**) VRSA48. P is a positive control without any antimicrobial agents, and N is a negative control with only media and DW. In the 96-well plate, from left to right, the GSE and pH 76% G (naringin) concentrations were 1/2 to 1/1024, and the concentrations of antibiotics were 32 to 0.06 µg/mL. The MICs of pH 76% G (naringin) are indicated by red arrows, and antibiotic MICs are indicated by blue arrows. MIC values <1/1024 or >32 µg/mL are not indicated by arrows. The MIC values for GSE, pH 76% G (naringin), and antibiotics for each of the bacterial strains are also illustrated by bar graphs next to the 96-well plate figures. In the bar graphs, the MIC values of GSE and pH 76% G (naringin) can be read along the left *y*-axis indicated by the red arrow, and the MIC values of antibiotics can be read along the right *y*-axis indicated by the blue arrow. GSE and the antibiotics showed the same effect, as shown in Figure 4. The pH 76% G (naringin) did not show a better effect than the pH 76% G. This indicates that substances other than naringin in GSE affect the antibacterial activity of GSE.

The antimicrobial activity of naringin against several bacterial species, including *S. aureus*, has already been confirmed in several papers [39,41,42,43]. The experiment was conducted after preparing a pH 76% G solution containing 1.7 mg/mL naringin. It is indicated as “pH 76% G (naringin)” in figures. In the 96-well plate figures, the MICs of pH 76% G and 76% G are indicated by red arrows, and antibiotic MICs are indicated by blue arrows. The MIC of GSE and the MICs for >32 µg/mL antibiotic are not indicated. In the bar graphs, the MICs of GSE and pH 76% G (naringin) can be read as values of the left *y*-axis (red arrow), and MICs of antibiotics can be read as values of the right *y*-axis (blue arrow). Compared to the results in Figure 4, GSE and antibiotics showed identical MIC values. The pH 76% G (naringin) also showed the same effects as pH 76% G without naringin against MSSA, MRSA 33591, MRSA 3795, and VRSA48. From the results of Figure 5, we knew that naringin did not play an important role in the antibacterial activity of GSE. The antibacterial effect of GSE can be exhibited by benzethonium chloride, a preservative commonly used in commercial GSE [44], but the GSE used in this experiment was confirmed by the manufacturer to be free of benzethonium chloride. Furthermore, the antimicrobial effect of GSE may result from synergistic effects of flavonoids [45]. However, because the concentrations of flavonoids other than naringin are quite small, the probability of a synergistic effect is very low. These our experiments and results can indirectly support the claim that the other phenolic compounds other than flavonoids in GSE contribute to the antimicrobial effects of GSE [46]. However, LC-MS/MS analysis was conducted without removing the solvent (glycerol) to find out the concentration of glycerol and to eliminate the potential risk that the filter to make concentrated GSE could filter out substances with antibacterial activity. Therefore, an experiment to analyze concentrated GSE is required, and then additional experiments to confirm the antibacterial activity of flavonoids and other phenolic compounds are required.

## 4. Conclusions

There are many papers on the antibacterial activity of GSE. They have shown antibacterial activity of GSE against MDR bacteria such as MRSA and VRE (vancomycin-resistant enterococci). However, the antibacterial effect of GSE on VRSA was first mentioned in this paper. GSE showed antibacterial activity against MSSA, MRSA, and VRSA in disk diffusion and microdilution MIC tests. The experiments were conducted with glycerol, acidic conditions, and naringin, which were expected to have antimicrobial activity, but the antibacterial effect of GSE was not sufficiently explained. Nevertheless, since GSE exerts favorable antimicrobial activity against not only MSSA but also MDR bacteria, it can be a valuable natural substance for preventing or reducing nosocomial infection, and further analysis is likely to be needed.

## Data Availability

Not applicable.

## Supplementary Materials

The following are available online at https://www.mdpi.com/2079-6382/10/1/85/s1, Figure S1: Antibacterial activity in 12 plant extract screening tests against MSSA; Figure S2. LC-MS Chromatogram of quercetin and hesperidin. LC–MS chromatograms showing; Figure S3. GSE incubation without bacteria and antimicrobial agents Table S1: List of plant extracts used for the antibacterial activity test.
