# Supplementary material for: Grapefruit Seed Extract as a Natural Derived Antibacterial Substance against Multidrug-Resistant Bacteria
Source: Antibiotics (Basel). 2021 Jan 18;10(1):85. doi: 10.3390/antibiotics10010085 (PMC7830962; doi:10.3390/antibiotics10010085)
Supplement: Supplementary file 1 [file antibiotics-10-00085-s001.pdf]

Supplementary data

Table S1. List of plant extracts used for the antibacterial activity test

Number	Plant extracts from	Extraction type	Source
1	Bamboo	Hot water	KoreaSimilac
2	Refined wood vinegar	Confidential	HERBFLORA
3	Rosemary	Hot water	HERBFLORA
4	*Pinus densiflora leaf*	Confidential	HERBFLORA
5	*Sophora*	Hot water	HERBFLORA
6	*Cinnamomum cassia bark*	Low temperature	HERBFLORA
7	*Hibiscus sabdariffa flower*	Low temperature	HERBFLORA
8	*Chamomilla recutita (Matricaria) flower*	Low temperature	HERBFLORA
9	*Centella asiatica*	Hot water	HERBFLORA
10	*Houttuynia cordata*	Hot water	HERBFLORA
11	Yucca	Hot water	HERBFLORA
12	Grapefruit seed	Confidential	CANDLEIKEA

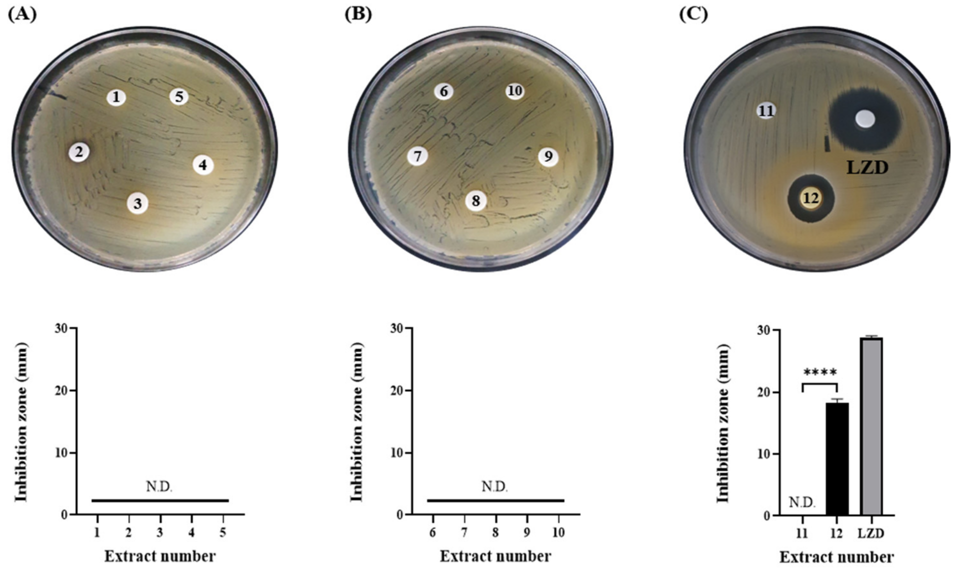

Figure S1. Antibacterial activity in 12 plant extract screening tests against MSSA. The experiment was conducted according to the extract number specified in Table S1. 1-5 (A), 6-10 (B), and 11-LZD (C). The concentration of LZD was 30 µg/disk. N.D. signifies that inhibition zone was not detected. The diameters of the inhibition zones are illustrated by the bar graphs as the means ± SD (n=3). LZD used as a quality control. Except for GSE, other plant extracts did not show antimicrobial effects against MSSA. Therefore, for subsequent experiments, only GSE was used. *p* values were calculated using two-tailed, unpaired *t*-tests in this figure. ****p <0.0001.

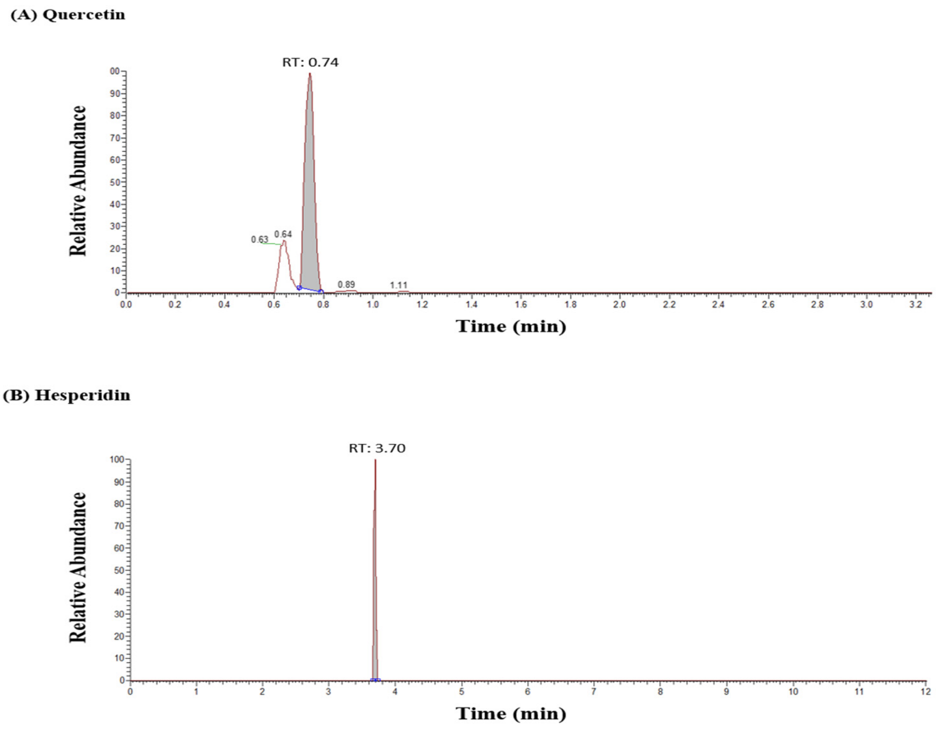

Figure S2. LC-MS Chromatogram of quercetin and hesperidin. LC–MS chromatograms showing (A) quercetin at 0.74 min and (B) hesperidin at 3.70 min. RT is retention time.

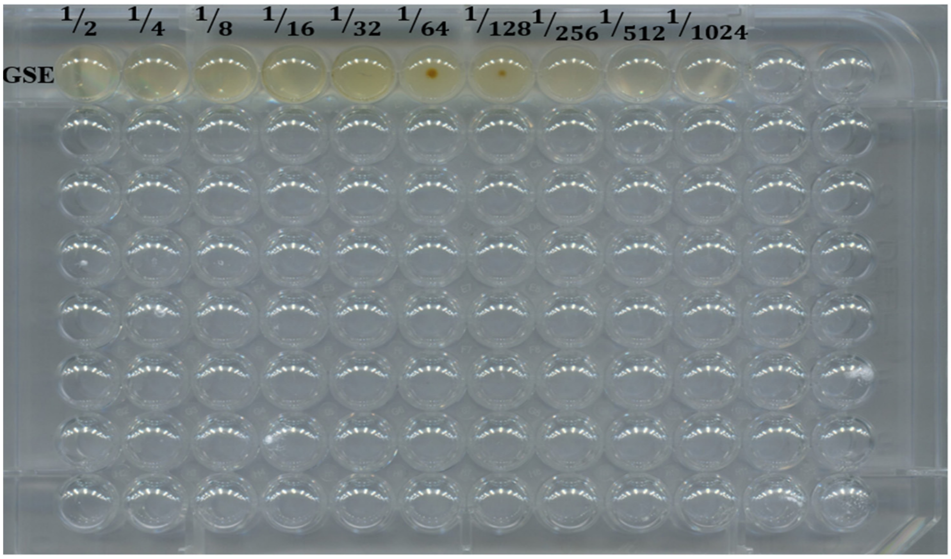

Figure S3. GSE incubation without bacteria and antimicrobial agents. From left to right, GSE concentrations are 1/2 to 1/1024. The purpose of this figure is to show the effect of GSE itself after incubation. This is the result of adding 100 μL of DW to 100 μL of diluted GSE from 1 to 1/512 concentration and incubating for 18 h at 37°C. Red dots were identified at 1/64 and 1/128 concentrations, and it was confirmed that the solution became cloudy at the surrounding concentrations.
